# Supplementary material for: Essentiality of fatty acid synthase in the 2D to anchorage-independent growth transition in transforming cells
Source: Nat Commun. 2019 Nov 1;10:5011. doi: 10.1038/s41467-019-13028-1 (PMC6825217; doi:10.1038/s41467-019-13028-1)

Supplementary Information

Essentiality of fatty acid synthase in the 2D to anchorage-independent growth transition in transforming cells

Bueno et al.

Supplementary Figure 1

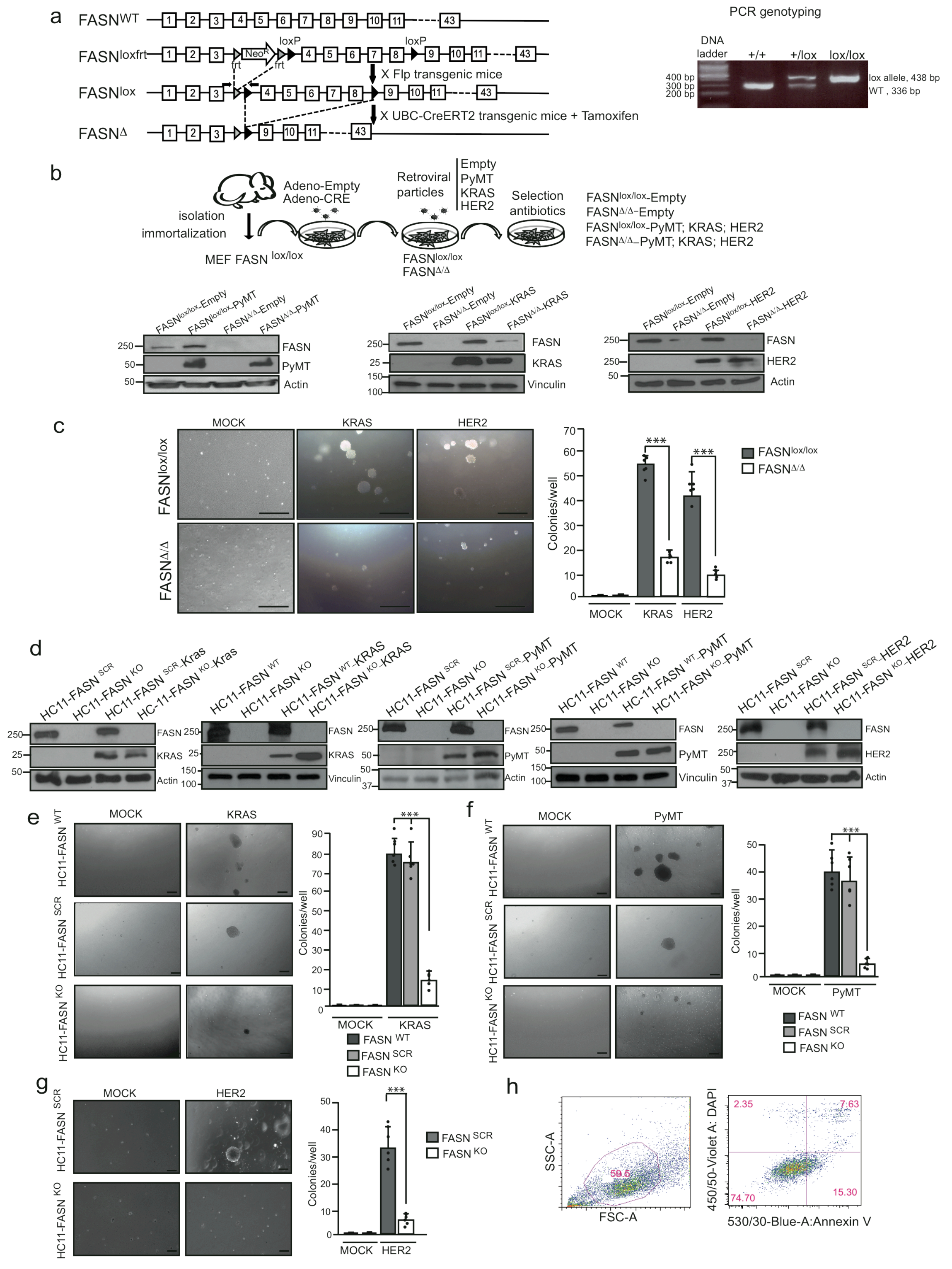

Supplementary Figure 1. FASN is essential for PyMT, KRAS, and HER2-mediated transformation. (a) Generation of FASN conditional knockout mice (left panel)

illustrating the target strategy of FASN. FASN wild-type ($FASN^{wt}$), floxed ($FASN^{lox}$), and conditionally deleted alleles ($FASN^{\Delta}$). In the conditional allele, exons 4–8 are flanked by loxP sites allowing for its deletion in the presence of Cre recombinase. Representative genotyping PCR results using specific set of primers to detect the presence of wt (336 bp) and lox alleles (438 bp) (right panel). **(b)** Scheme of *in vitro* transformation protocol. Mouse embryonic fibroblast (MEF) cells were derived from E12.5 $FASN^{lox/lox}$ embryos and immortalized with SV40 T antigen. Cre/empty adenoviral infection was performed 3 days before infection with the PyMT, KRAS (G12D), or HER2 (A775_G776insYVMA) oncogene-containing or empty retroviruses. Western blotting analysis of FASN, KRAS (G12D), HER2 (A775_G776insYVMA), and PyMT protein levels in MEFs isolated from $FASN^{lox/lox}$ animals one week after infection are shown. **(c)** $FASN^{lox/lox}$-KRAS/HER2 and $FASN^{\Delta/\Delta}$-PyMT/KRAS/HER2 MEFs were plated in soft agar as described in Methods. After 3-4 weeks, colonies were quantified per well (n=6 per genotype). Photos show representative fields from each genotype. Scale bars, 500 μm. Presented data are the mean values ± SD. ***P < 0.001; Student's t test. Experiment was repeated three times. **(d)** HC11 murine breast epithelial cells infected with SgRNA against mock sequences (SCR) or FASN (KO) were infected with empty, KRAS (G12D), PyMT or HER2-containing retroviruses; FASN, KRAS, PyMT and HER2 protein levels are shown. Soft-agar colony formation assays of HC11 cells (WT, SCR and KO for FASN) in the presence of KRAS **(e)**, PyMT **(f)** and HER2 **(g)** are shown. Representative pictures of each genotype are shown in the panels on the left and the colony quantification charts are shown on the right (n=6 wells per genotype). Scrambled (SCR) clones showed the same phenotype than the WT cells. Scale bars, 500 μm. **(h)** Representative example of gating strategy used to characterize $FASN^{lox/lox}$-PyMT MEFs in apoptosis in 2D cultures, using co-staining with Annexin-V and DAPI. Presented data are the mean values ± SD of three independent experiments. ***P < 0.001; unpaired Student's t test.

Supplementary Figure 2

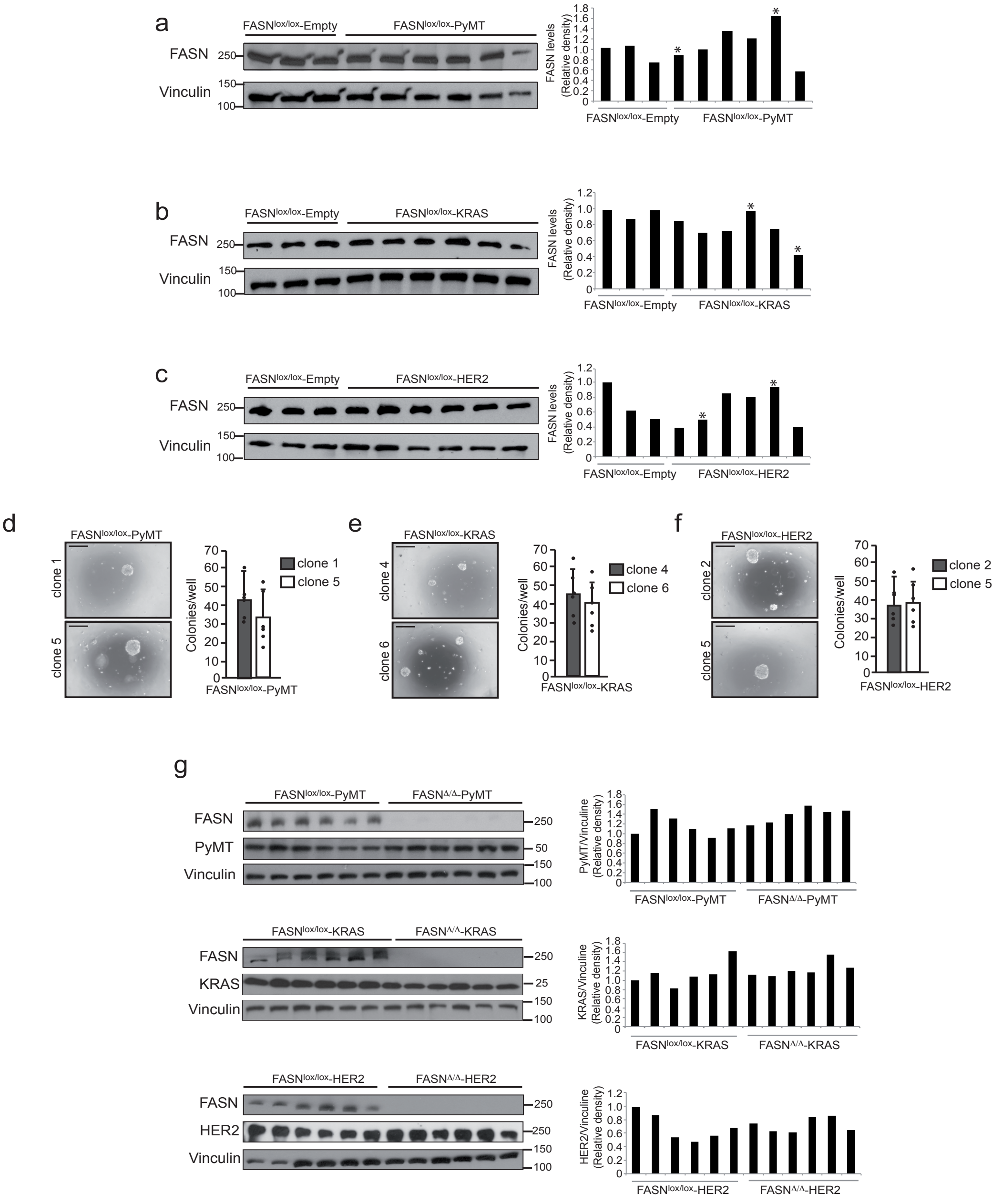

Supplementary Figure 2. Minor variations of FASN levels do not impact in the clonogenic ability of PyMT, KRAS and HER2 oncogenes. Western blots for FASN in non-transformed (FASN$^{lox/lox}$-Empty) versus transformed clones (FASN$^{lox/lox}$-PyMT **(a)**; FASN$^{lox/lox}$-KRAS **(b)** and FASN$^{lox/lox}$-HER2 **(c)**. Quantitative analysis of the western blots results is shown (right charts). FASN levels displayed minor fluctuations of similar magnitude both in transformed and non-transformed clones. Soft-agar colony assays performed picking clones with the greatest differences in FASN levels [marked by asterisks in panels **(a)**, **(b)** and **(c)**] showed equally proficient colony formation capacity in the experiments shown in panels **(d)** – PyMT - **(e)** – KRAS – or **(f)** – HER2. Representative images of the soft agar assay (left panel) and quantitation of colonies per well recovered from each genotype (right panel) are shown. Scale bars, 500 μm. Presented data are the mean values ± SD. Student's *t* test. The experiment was repeated three times. **(g)** Western blots for PyMT, KRAS and HER2 levels across different FASN$^{lox/lox}$ and FASN$^{\Delta/\Delta}$ clones did not reveal significant differences. Quantitative analysis of the western blots results is shown (right charts).

Supplementary Figure 3

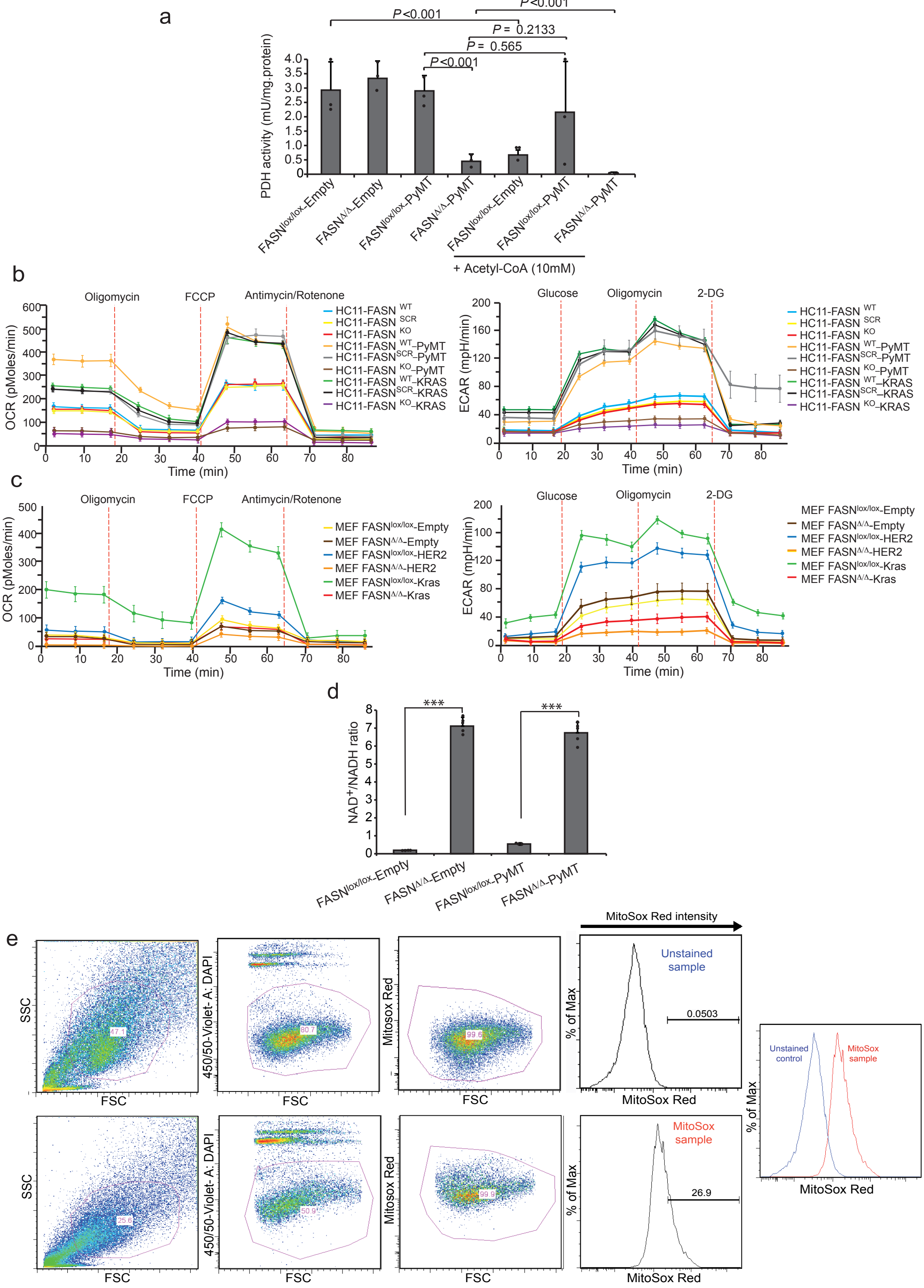

Supplementary Figure 3. Metabolic phenotype caused by FASN deletion before transformation. (a) Pyruvate dehydrogenase activity observed across the 4 genotypes in isolated mitochondria from fresh cells. The addition of exogenous acetyl-CoA (10 mM) directly to the mitochondrial lysates induced a decrease in pyruvate dehydrogenase activity in FASN-positive MEFs (FASN$^{lox/lox}$ MEFs). Presented data are the mean values ± SD. Student's *t* test was performed. Experiment was repeated three times. **(b)** Inhibition of maximum respiratory capacity and glycolysis. Representative oxygen consumption rate levels (OCR) (left panel) and extracellular acidification rate (ECAR) (right panel) charts observed in HC11 murine breast epithelial cells (FASN-positive, HC11-FASN$^{WT,\ SCR}$; and FASN-negative, HC11-FASN^{KO} cells) transformed with PyMT or KRAS. Data are represented as mean ± SEM. Experiments were repeated 3 times for each genotype. **(c)**. Same as **(b)**, but with FASN$^{lox/lox}$ and FASN$^{\Delta/\Delta}$ MEFs transformed with HER2 or KRAS. **(d)** In the absence of FASN (FASN$^{\Delta/\Delta}$-Empty and FASN$^{\Delta/\Delta}$-PyMT), NAD^+/NADH quotients are reverted, showing a decreased capacity of NAD^+ regeneration (n=5). Presented data are the mean values ± SD. ***P < 0.001; unpaired Student's *t* test. **(e)** Representative flow cytometry graphs showing the gating strategy for measuring ROS levels.

Supplementary Figure 4

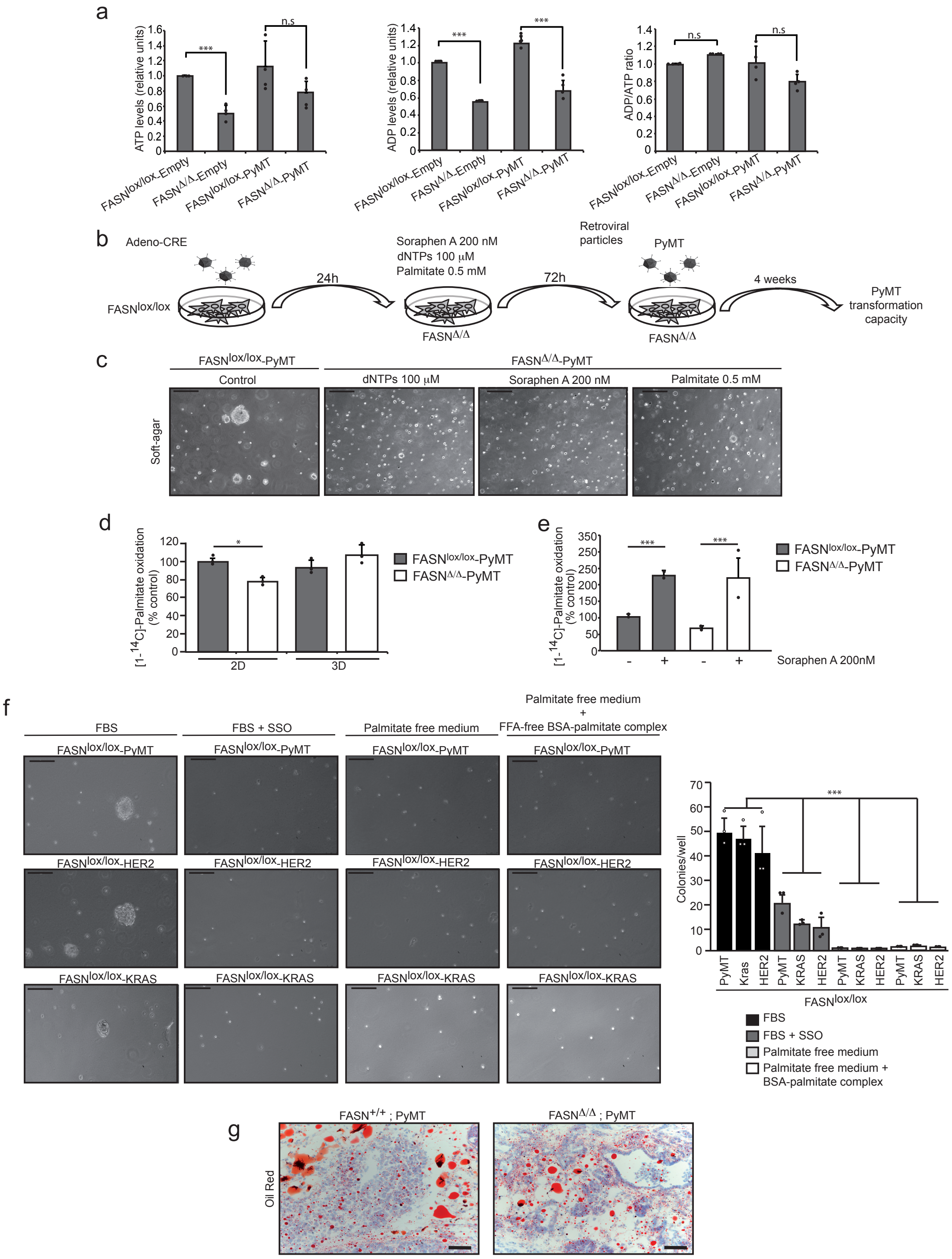

Supplementary Figure 4. ADP/ATP quotients and lack of phenotype reconstitution by supplementations. (a) ATP and ADP levels were measured using ATP and ADP determination kits. Presented data are the mean values ± SD of four independent experiments. ***P < 0.001, n.s: not significant; Student's *t* test. **(b)** Scheme depicting the timepoints of the phenotype rescue experiments: soraphen A, dNTPs, and/or palmitate were added to tissue culture media after FASN deletion and before transformation. **(c)** The addition of dNTPs, soraphen A and palmitate does not rescue the soft-agar colony formation ability of $FASN^{\Delta/\Delta}$-PyMT MEFs (n=6 cultures from a representative experiment). Representative pictures are shown. Scale bars, 500 μm. **(d)** Palmitate oxidation was assessed by adding [1-^{14}C]palmitate to the media and measuring its incorporation into CO_2 (n=3 cultures per genotype) in $FASN^{lox/lox}$-PyMT versus $FASN^{\Delta/\Delta}$-PyMT MEFs in monolayer cultures (2D) or spheroid cultures (3D). Presented data are the mean values ± SD of three independent experiments. *P < 0.05; Student's *t* test. **(e)** Palmitate oxidation was assessed in presence of soraphen A in $FASN^{lox/lox}$-PyMT *versus* $FASN^{\Delta/\Delta}$-PyMT MEFs in monolayer cultures (n=3 culture per genotype). Presented data are the mean values ± SD of two independent experiments. ***P < 0.001; Student's *t* test. **(f)** Soft-agar colony assays of $FASN^{lox/lox}$-PyMT, -HER2, - and -KRAS MEFs in the presence of culture medium with FBS (left) (palmitate represents 26% of the fatty acid pool of regular pools[6]). In addition, $FASN^{lox/lox}$-PyMT, -HER2 or -KRAS MEFs were incubated in full medium with FBS and the fatty acid uptake inhibitor sulfo-N-succinimidyl oleate (SSO)[7]. Finally, fatty acid-free media was supplemented with FFA-free-BSA-palmitate complexed. Representative pictures are shown. Colony quantification charts are shown on the right (n=6 wells per genotype and condition). Scale bars, 500 μm. Presented data are the mean values ± SD of three independent experiments. ***P < 0.001; unpaired Student's *t* test. **(g)** Positive Oil Red staining of tumor epithelium from $FASN^{+/+}$; PyMT and $FASN^{\Delta/\Delta}$; PyMT animals. A representative image per genotype is shown. Scale bars, 50 μm.

Supplementary Figure 5

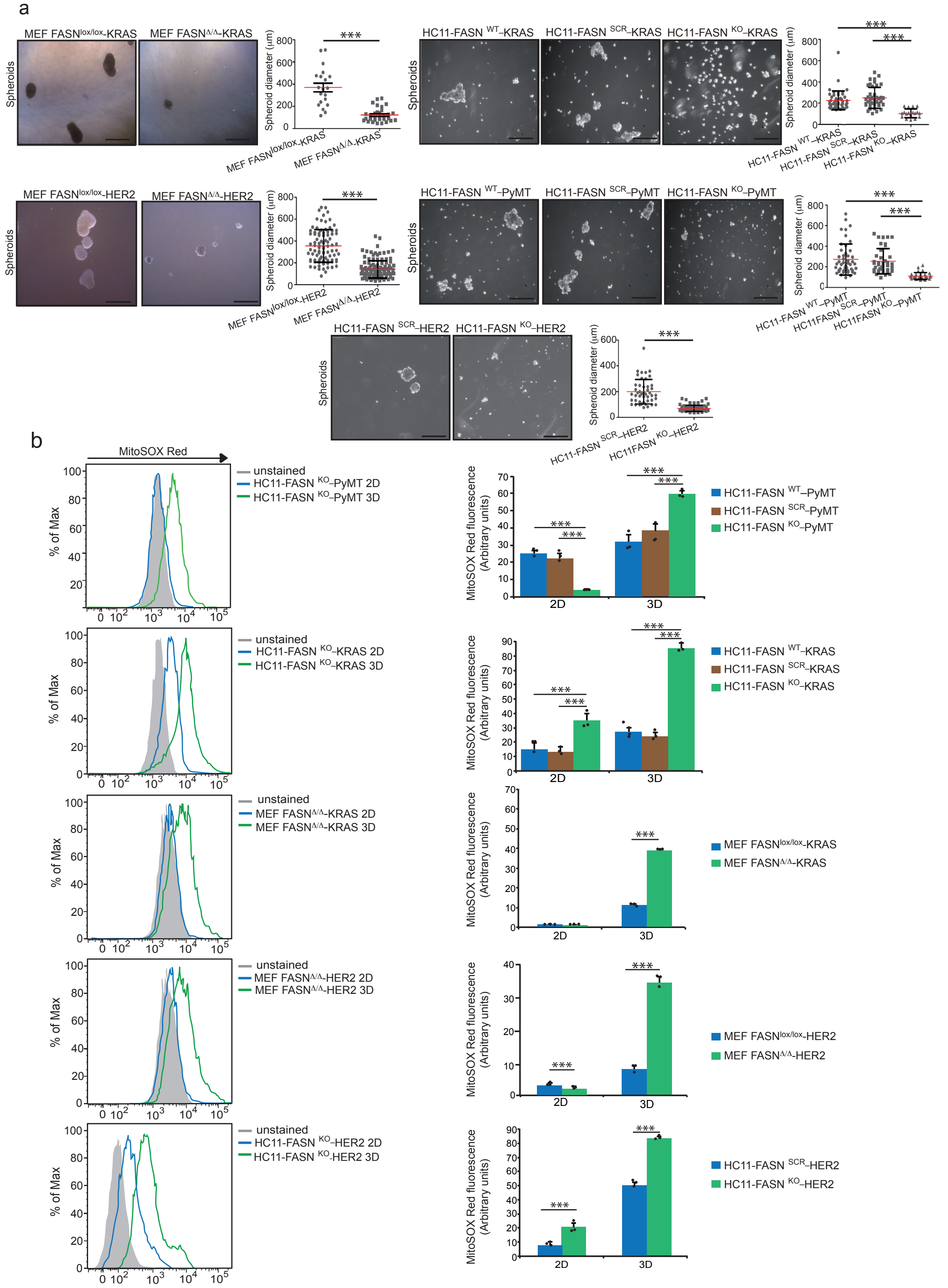

Supplementary Figure 5. 3D growth inhibition and increased ROS in 3D growth compared with 2D in absence of FASN. (a) Representative pictures of spheroids recovered from HC11 cells expressing the KRAS (G12D) (upper right), PyMT (middle right) or HER2 (lower right) oncogenes, wild-type (WT), SCR (scrambled) or KO for FASN, growing under ultra-low attachment conditions (n=6 cultures per genotype). Average spheroid diameters are depicted in the right panels (HC11-FASN^{WT}-KRAS, n= 48 spheroids; HC11-FASN^{SCR}-KRAS, n= 41 spheroids; HC11-FASN^{ko}-KRAS, n= 18 spheroids; HC11-FASN^{WT}-PyMT, n= 48 spheroids; HC11-FASN^{SCR}-PyMT, n= 40 spheroids; HC11-FASN^{ko}-PyMT, n= 30 spheroids; HC11-FASN^{SCR}-HER2, n= 46 spheroids; HC11-FASN^{ko}-HER2, n= 71 spheroids). MEFs expressing mutant KRAS (upper left) or HER2 (lower left) showed similar behavior (n=6 culture for each genotype; FASN$^{lox/lox}$ –KRAS n=20 spheroids; FASN$^{\Delta/\Delta}$ -KRAS n=31 spheroids; FASN$^{lox/lox}$ –HER2 n=90 spheroids; FASN$^{\Delta/\Delta}$ -HER2 n=130 spheroids). In all cases, scrambled (SCR) clones showed the exact same phenotype than the WT cells. Scale bars, 500 μm. Presented data are the mean values ± SD. ***P < 0.001; Student's t test. Experiments were repeated 3 times. **(b)** ROS levels. Left panels: representative flow cytometry analysis showing constitutive mitochondrial superoxide levels in 2D (monolayer) compared with 3D (spheroids) conditions in HC11-FASN^{KO}-PyMT, HC11-FASN^{KO}-KRAS, HC11-FASN^{KO}-HER2, MEF FASN$^{\Delta/\Delta}$-KRAS and MEF FASN$^{\Delta/\Delta}$-HER2 (n=6 cultures for each condition). Right panels: quantification of MitoSOX red fluorescence intensity. MitoSOX red fluorescence intensity increases dramatically in FASN-negative HC11 and MEFs when they express an oncogene (PyMT, KRAS (G12D), or HER2) and are challenged to grow in 3D compared with 2D. However, FASN-positive cells show a significantly smaller increase in ROS levels from 2D to 3D. In all cases, scrambled (SCR) clones showed the exact same phenotype than the WT cells. Presented data are the mean values ± SD of three independent experiments. ***P < 0.001; unpaired Student's t test.

Supplementary Figure 6

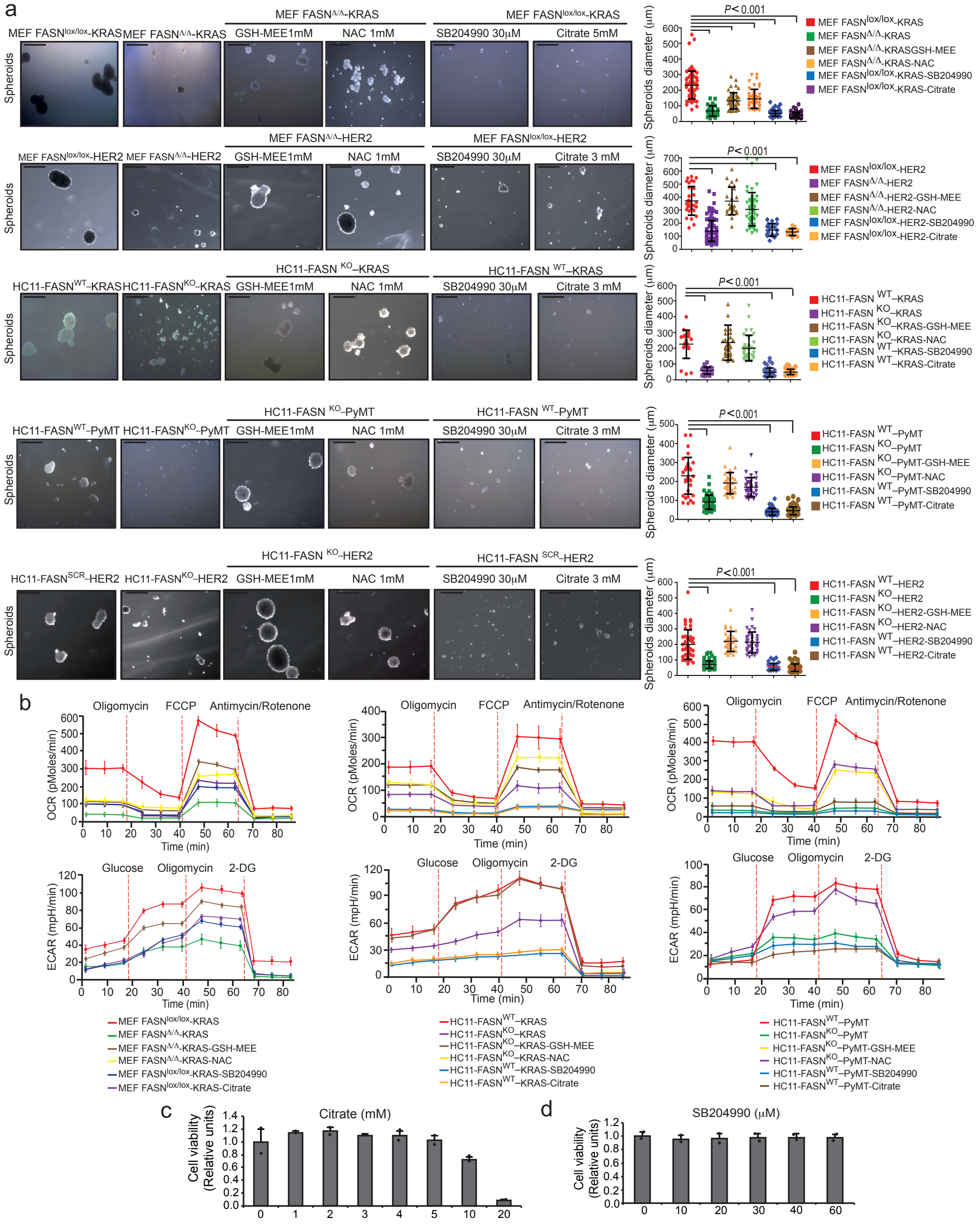

Supplementary Figure 6. Phenotype rescue in FASN-negative cells and phenocopy in FASN-positive cells. Phenotype rescue by NAC/GSH-MEE. NAC (1 mM) and GSH-MEE (1 mM) could rescue spheroid formation **(a)** and OCR and ECAR

levels **(b)** in FASN$^{\Delta/\Delta}$-KRAS and FASN$^{\Delta/\Delta}$-HER2 MEFs, and HC11-^{KO}-KRAS, HC11-^{KO}-PyMT and HC11-^{KO}-HER2 cells similar to those observed in FASN wt cells overexpressing those oncogenes (MEF FASN$^{lox/lox}$-KRAS, MEF FASN$^{lox/lox}$-HER2, HC11-^{WT}-KRAS, HC11-^{WT}-PyMT and HC11-^{SCR}-HER2 respectively). In contrast, sodium citrate (3mM) or SB204990 (ATP citrate lyase inhibitor) (30 μM) treatment in FASN-positive cells before oncogenes infections was accompanied by a decreased in OCR and ECAR levels **(b)** and disruption of spheroid formation **(a)** at a level similar to that observe in FASN-negative cells; FASN$^{\Delta/\Delta}$-KRAS MEFs, FASN$^{\Delta/\Delta}$-HER2 MEFs, HC11-^{KO}-KRAS, HC11-^{KO}-PyMT and HC11-^{KO}-HER2**. (a)** Representative pictures of the spheroid cultures (left) (n=6 culture per genotype and condition) and spheroid diameter quantification (right) are shown (MEF FASN$^{lox/lox}$-KRAS, n=73 spheroids; FASN$^{\Delta/\Delta}$-KRAS, n=29; FASN$^{\Delta/\Delta}$-KRAS-GSH-MEE, n=55; FASN$^{\Delta/\Delta}$-KRAS-NAC, n=57; FASN$^{lox/lox}$-KRAS-SB204990, n=20; FASN$^{lox/lox}$-KRAS-citrate, n=82; MEF FASN$^{lox/lox}$-HER2, n=32; FASN$^{\Delta/\Delta}$- HER2, n=30; FASN$^{\Delta/\Delta}$- HER2-GSH-MEE, n=28; FASN$^{\Delta/\Delta}$- HER2-NAC, n=57; FASN$^{lox/lox}$- HER2-SB204990, n=20; FASN$^{lox/lox}$- HER2-citrate, n=13; HC11-FASN^{WT} –KRAS, n= 21; HC11-FASN^{KO} –KRAS, n= 29: HC11-FASN^{KO} –KRAS-GSH-MEE, n=31; HC11-FASN^{KO} –KRAS-NAC, n=35; HC11-FASN^{WT} –KRAS-SB204990, n=60; HC11-FASN^{WT} –KRAS-citrate, n=51; HC11-FASN^{WT} –PyMT, n= 33; HC11-FASN^{KO} – PyMT, n= 53: HC11-FASN^{KO} – PyMT -GSH-MEE, n=32; HC11-FASN^{KO} – PyMT -NAC, n=32; HC11-FASN^{WT} – PyMT -SB204990, n=47; HC11-FASN^{WT} – PyMT -citrate, n=69; HC11-FASN^{SCR} –HER2, n= 46; HC11-FASN^{KO} – PyMT, n= 71; HC11-FASN^{KO} – HER2 -GSH-MEE, n=36; HC11-FASN^{KO} – HER2 -NAC, n=42; HC11-FASN^{SCR} – HER2 -SB204990, n=36; HC11-FASN^{SCR} – HER2 -citrate, n=38). Presented data are the mean values ± SD. $P < 0.001$; unpaired Student's *t* test. Scale bars, 500 μm. **(c)** Cell viability in HC11 cells during 72 hours in the presence of various concentrations of sodium citrate or **(d)** SB204990 in the media under 2D culture conditions. Presented data are the mean values ± SD of two independent experiments (n=3 experimental replicates).

Supplementary Figure 7

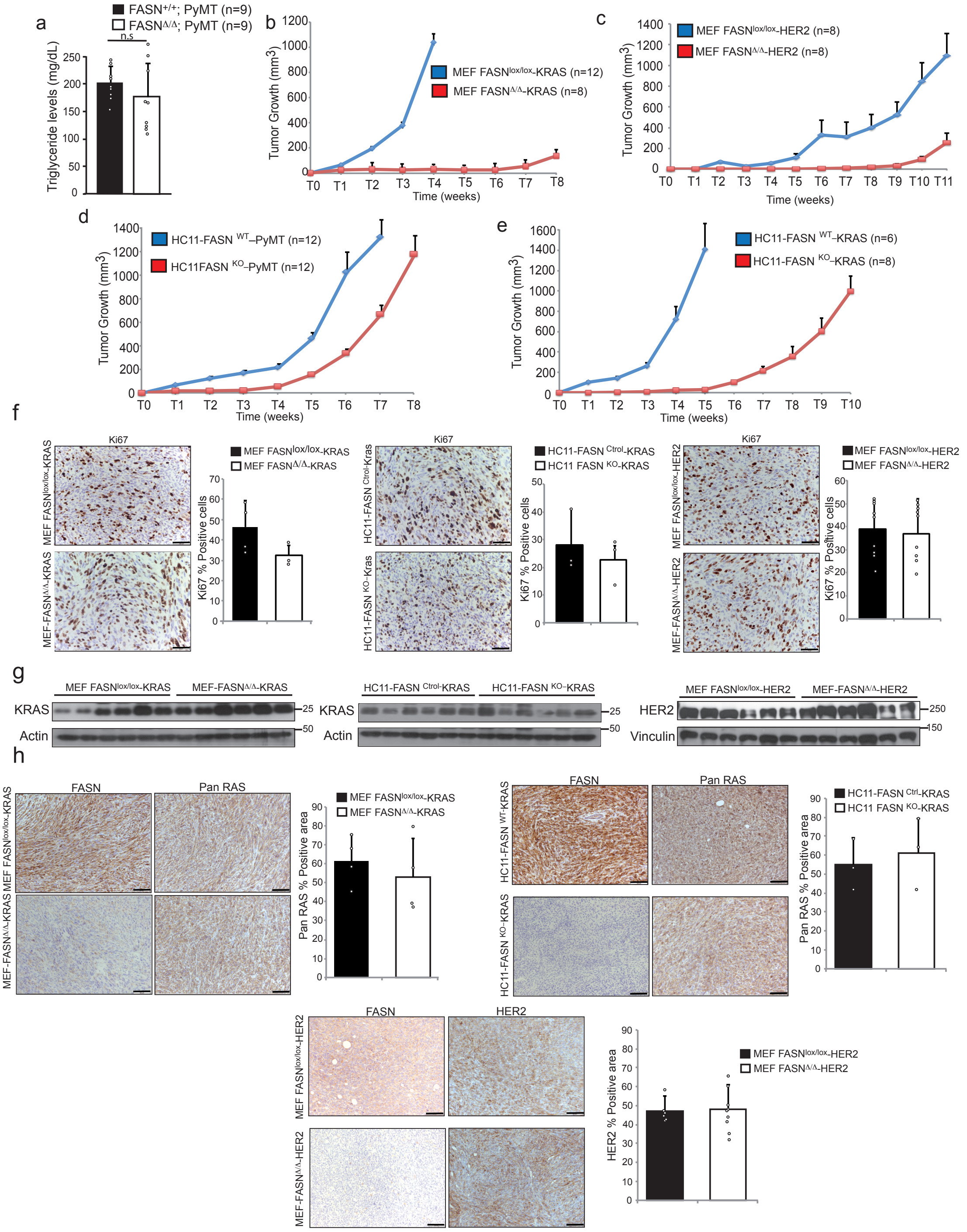

Supplementary Figure 7. Tumor growth inhibition in FASN-negative systems expressing various oncogenes. (a) Blood triglyceride levels of fasted FASN$^{lox/lox}$; PyMT and FASN$^{\Delta/\Delta}$; PyMT mice were measured at the time of sacrifice; no significant differences were observed among genotypes. Presented data are the mean values ± SD (n=9 mice per genotype). Panels **(b)** to **(e)** show tumor growth curves of mice with intramammary injections of cells with the different genotypes. FASN$^{\Delta/\Delta}$ MEFs infected with KRAS (MEF FASN$^{\Delta/\Delta}$-KRAS) **(b)** or HER2 (MEF FASN$^{\Delta/\Delta}$-HER2) **(c)** present a delay in tumor formation when grafted into wild-type animals compared with FASN$^{lox/lox}$ counterparts. Similarly, HC11-KO FASN cells present a delay in tumor formation when grafted into wild-type animals despite infection with PyMT (HC-11-^{KO}-PyMT) **(d)** or KRAS (HC-11-^{KO}-KRAS) **(e)**, compared with wild-type counterparts. Cells were injected into the left and right flank of nude mice. Points represent the mean tumor volume ± SD at each time point (weeks). **(f)** Ki67 staining in tumors originated from FASN$^{lox/lox}$ or FASN$^{\Delta/\Delta}$ clones and HC11^{Ctrol} or HC11^{KO} FASN cells reveals no changes in replication levels. Representative images are shown. Scale bars, 100 μm. Zen software (Zeiss Microscopy) was used to quantify positive cells for Ki67 staining (MEF FASN$^{lox/lox}$-KRAS, n=4 tumors; MEF FASN$^{\Delta/\Delta}$ KRAS, n=3 tumors; HC11^{Ctrol} –KRAS, n=3 tumors; HC11^{KO}–KRAS, n=3 tumors; MEF FASN$^{lox/lox}$-HER2, n=9 tumors; MEF FASN$^{\Delta/\Delta}$ HER2, n=9 tumors). Presented data are the mean values ± SD. No significant differences; Student's *t* test. KRAS and HER2 levels by WB **(g)** and IHQ **(h)** were similar in tumors arising from FASN$^{lox/lox}$ and FASN$^{\Delta/\Delta}$ clones and HC11^{Ctrol} or HC11^{KO} FASN cells. **(h)** Zen software (Zeiss Microscopy) was used to quantify positive area for KRAS and HER2 staining (MEF FASN$^{lox/lox}$-KRAS, n=4 tumors; MEF FASN$^{\Delta/\Delta}$ -KRAS, n=4 tumors; HC11^{Ctrol} –KRAS, n=4 tumors; HC11^{KO} –KRAS, n=4 tumors; MEF FASN$^{lox/lox}$-HER2, n=6 tumors; MEF FASN$^{\Delta/\Delta}$ HER2, n=9 tumors). Presented data are the mean values ± SD. Not significant differences; unpaired Student's *t* test.

Supplementary Figure 8

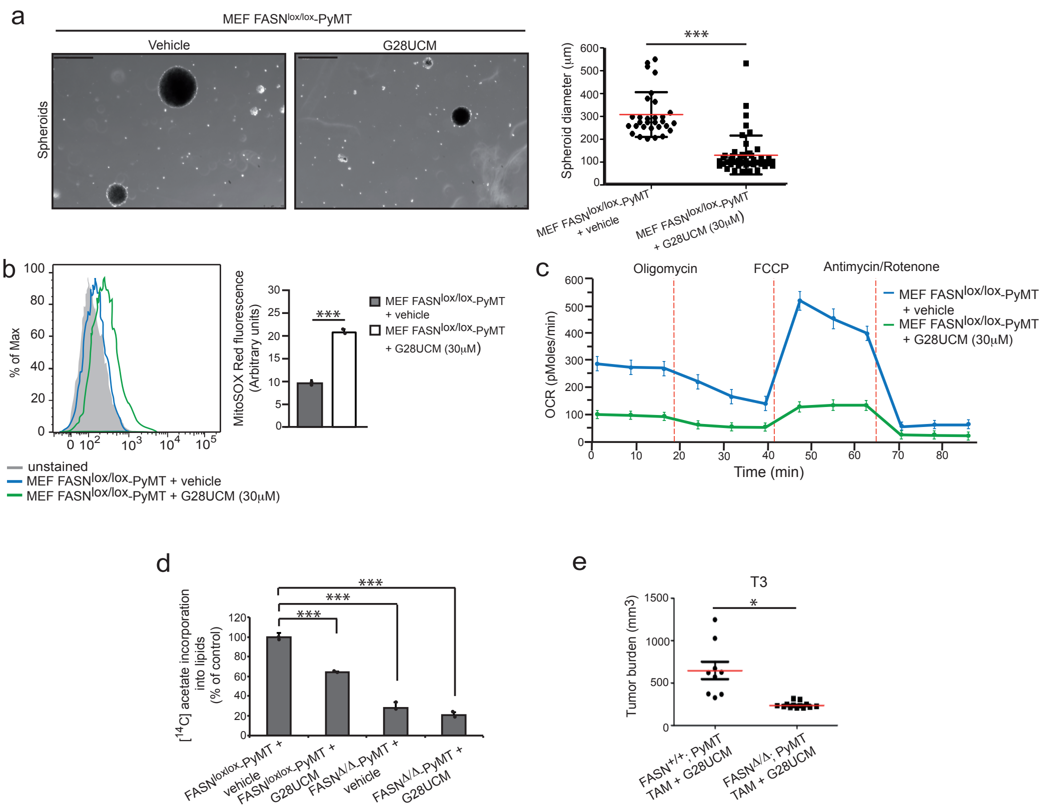

Supplementary Figure 8. G28UCM recapitulates most of the features of the FASN$^{\Delta/\Delta}$-PyMT MEFs. (a) Spheroid formation capacity of MEF FASN$^{lox/lox}$-PyMT significantly diminished in the presence of G28UCM FASN inhibitor (30μM) after 72h. Representative images from FASN$^{lox/lox}$-PyMT+ vehicle and FASN$^{lox/lox}$-PyMT+G28UCM are shown. Scale bars, 500 μm. Spheroid diameter quantification on the right (FASN$^{lox/lox}$-PyMT+vehicle, n=36 spheroids; FASN$^{lox/lox}$-PyMT+G28UCM, n= 47 spheroids). Presented data are the mean values ± SD. ***P < 0.001; unpaired Student's t test. **(b)** Representative flow cytometry analysis showing constitutive mitochondrial superoxide levels in 3D in FASN$^{lox/lox}$-PyMT+vehicle compared with FASN$^{lox/lox}$-PyMT+G28UCM MEFs loaded with MitoSOX Red (3μM). The quantification of MitoSOX Red fluorescence intensity is shown on the right (n=6 cultures for each

condition). Represented data are the mean values ± SD of two independent experiments. ***$P < 0.001$; Student's t test. **(c)** OCR rate decrease in $FASN^{lox/lox}$-PyMT MEFs treated with G28UCM in comparation with vehicle treated MEFs. Represented data are the mean values ± SEM of three independent experiments. **(d)** Fatty acid synthesis was assessed by adding ^{14}C-acetate to the media and measuring its incorporation into lipid in $FASN^{lox/lox}$-PyMT *versus* $FASN^{\Delta/\Delta}$-PyMT MEFs, in presence or absence of G28UCM in monolayer cultures (n=6 cultures per genotype and condition). Presented data are the mean values ± SD of two independent experiments. ***$P < 0.001$; Student's t test. Experiment was repeated twice. **(e)** *In vivo* effects of combination of pharmacologic inhibition (G28UCM) and genetic ablation (tamoxifen) of FASN. Tumor burden chart at T3 (3 weeks since treatment initiation) decreases in $FASN^{\Delta/\Delta}$; PyMT mice when they received G28UCM treatment (n=10 mice) compared with vehicle (n=7 mice). Presented data are the mean values ± SEM. *$P < 0.05$; unpaired Student's t test.

Supplementary Figure 9

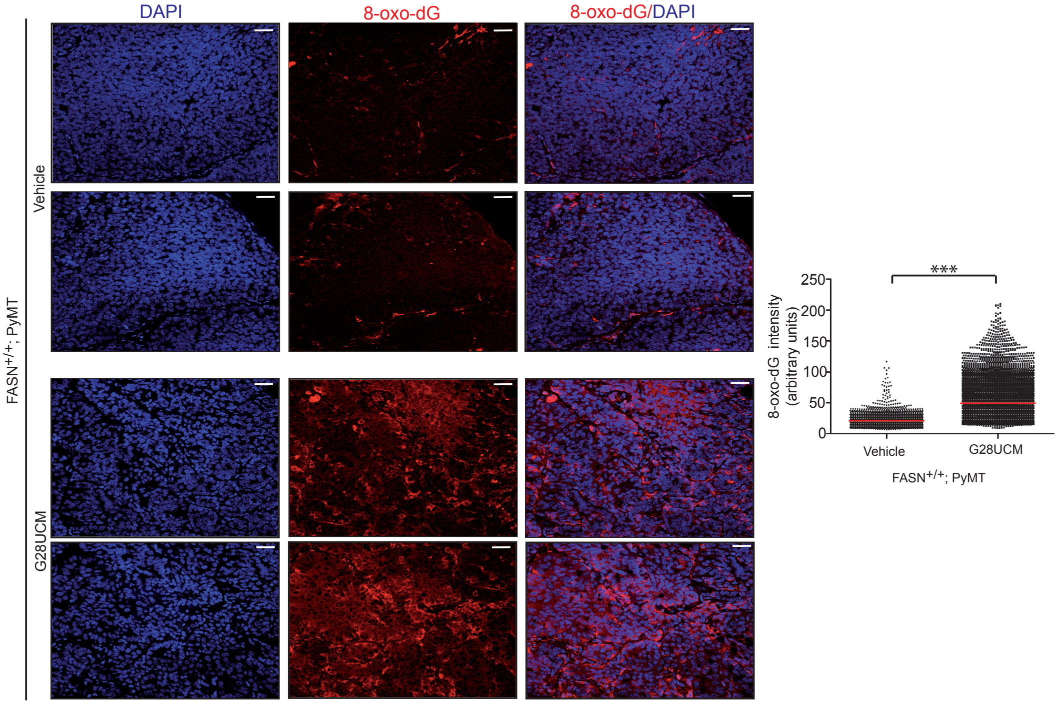

Supplementary Figure 9. G28UCM induces high ROS levels in FASN+/+; PyMT animals. Representative fluorescent images of DAPI (blue) and 8-oxo-deoxyguanosine (8-oxo-dG (red) staining of mammary pad sections of FASN$^{+/+}$; PyMT animals treated with vehicle or G28UCM obtained by immunofluorescence confocal microscopy. Merged images are shown. Scale bars, 50μm. The quantification of cellular 8-oxo-dG fluorescence intensity is shown on the right (n=5061 cells for FASN$^{+/+}$; PyMT + vehicle and n=9648 cells for FASN$^{+/+}$; PyMT + G28UCM were examined). Presented data are the mean values ± SD. ***P < 0.001; unpaired Student's *t* test.

Supplementary Figure 10

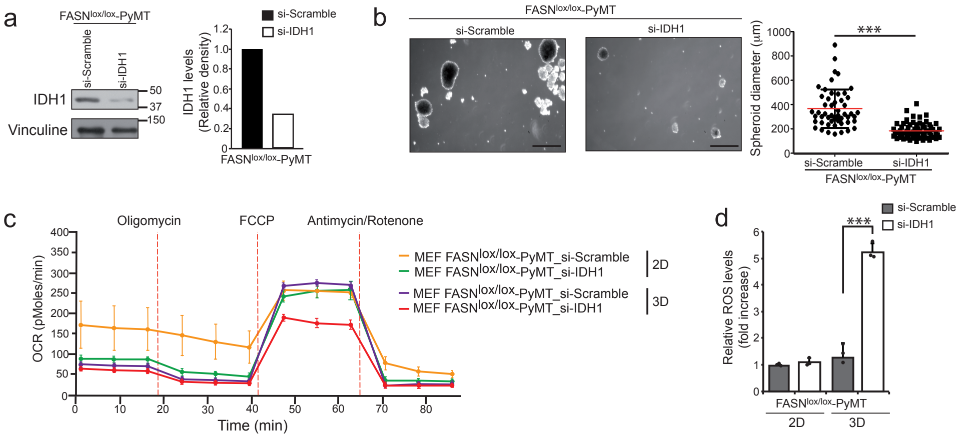

Supplementary Figure 10. IDH1 silence phenocopies the main features of FASN deletion in 3D. (a) Western blot of IDH1 in FASN$^{lox/lox}$-PyMT MEFs 72h after siRNA transfection. Quantitative analysis of the western blot results is shown (right chart). **(b)** Representative pictures of spheroids recovered from the previous experiment. IDH1 knockdown significantly decreases spheroid diameter in FASN$^{lox/lox}$-PyMT MEFs (FASN$^{lox/lox}$-PyMT-si-IDH1, n=63 spheroids; FASN$^{lox/lox}$-PyMT-si-Scramble, n=37 spheroids). Represented data are the mean values ± SD. ***P < 0.001; unpaired Student's t test. Scale bars, 500 μm. **(c)** Mitochondrial respiration reflected by oxygen consumption rate levels (OCR) was detected in FASN$^{lox/lox}$-PyMT-si-Scramble and FASN$^{lox/lox}$-PyMT-si-IDH1 MEFs under 2D and 3D culture conditions (n=12 experimental replicates per genotype, two independent experiments). Data are represented as mean values ± SEM. **(d)** Staining of FASN$^{lox/lox}$-PyMT MEFs (monolayer (2D) or spheroid (3D) cultures containing or lacking IDH1 with the mitochondrial ROS sensor MitoSox (n=3 cultures per condition). Represented data are the mean values ± SD. ***P < 0.001; unpaired Student's t test.

Supplementary Figure 11. Uncropped gels

Figure 1b

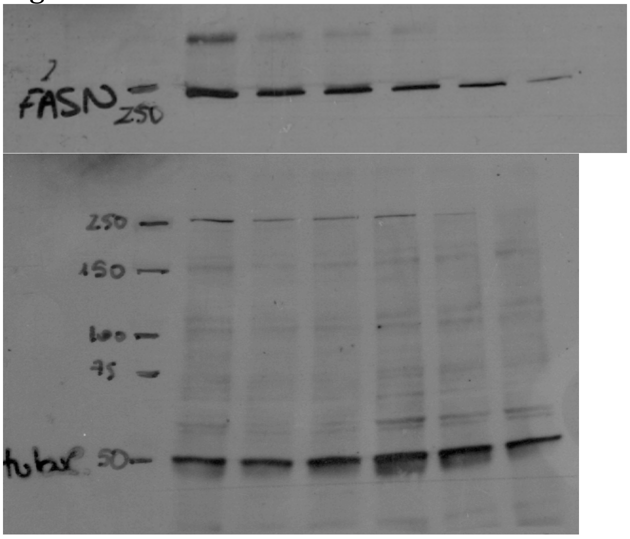

Supplementary Figure 1b

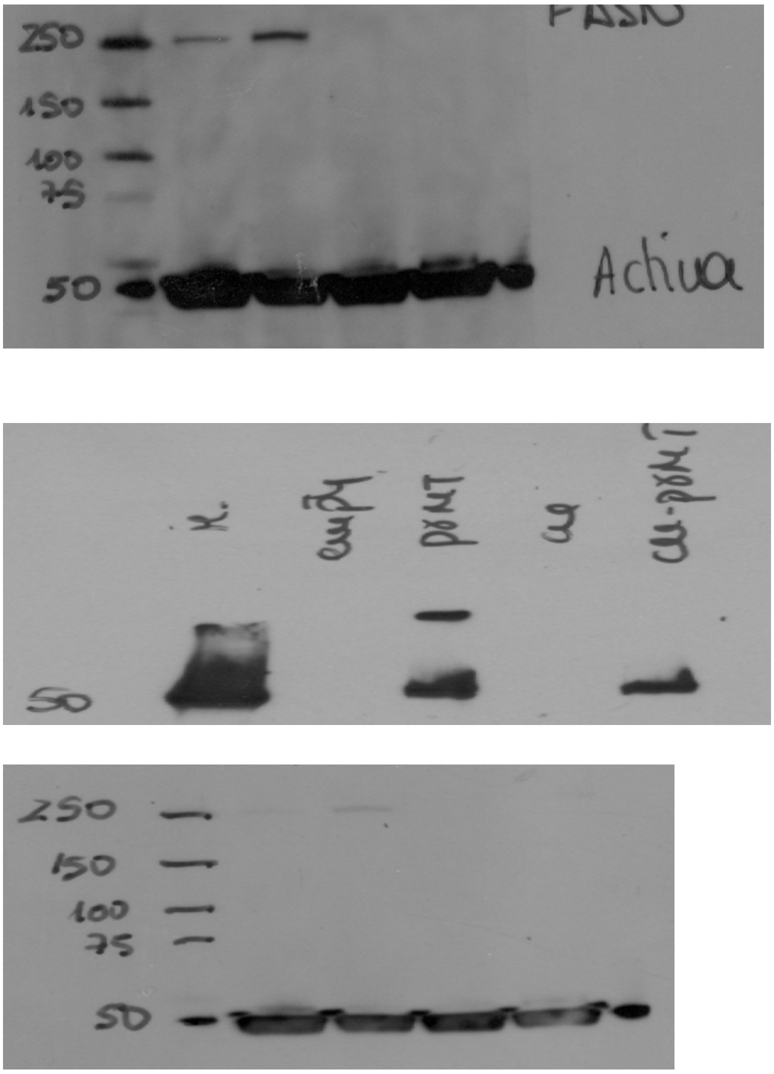

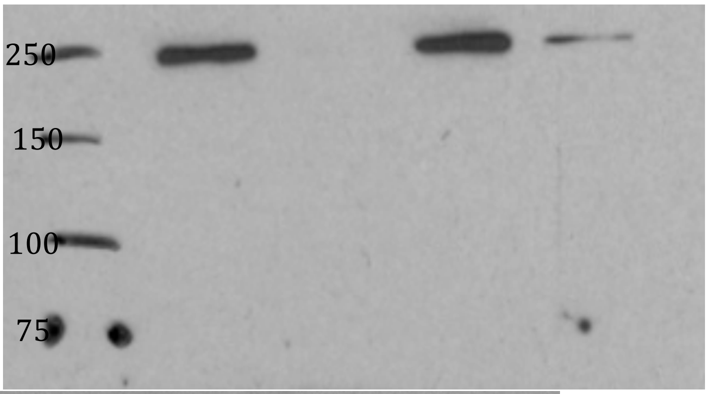
250
150
100
75

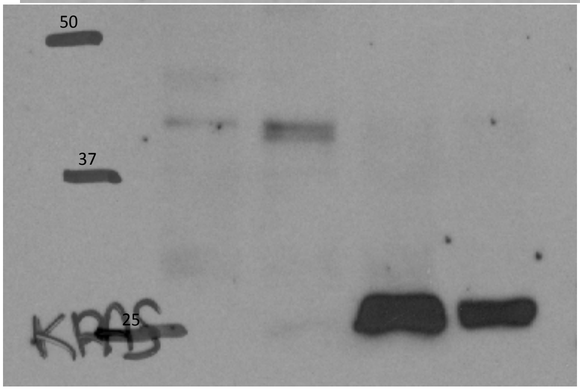
50
37
KRAS
25

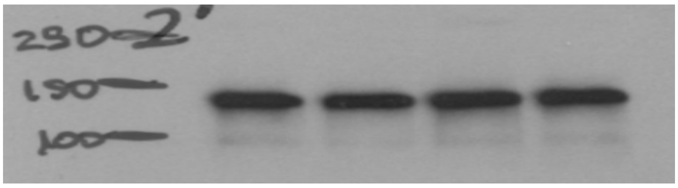
250
2'
150
100

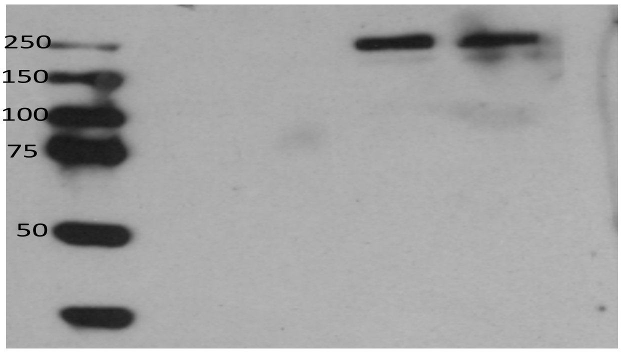
250
150
100
75
50

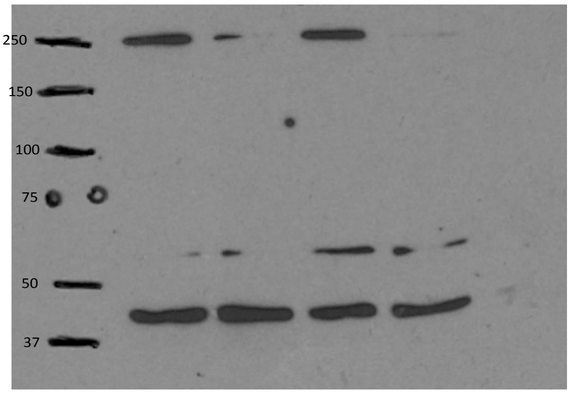
250
150
100
75
50
37

Supplementary 1d

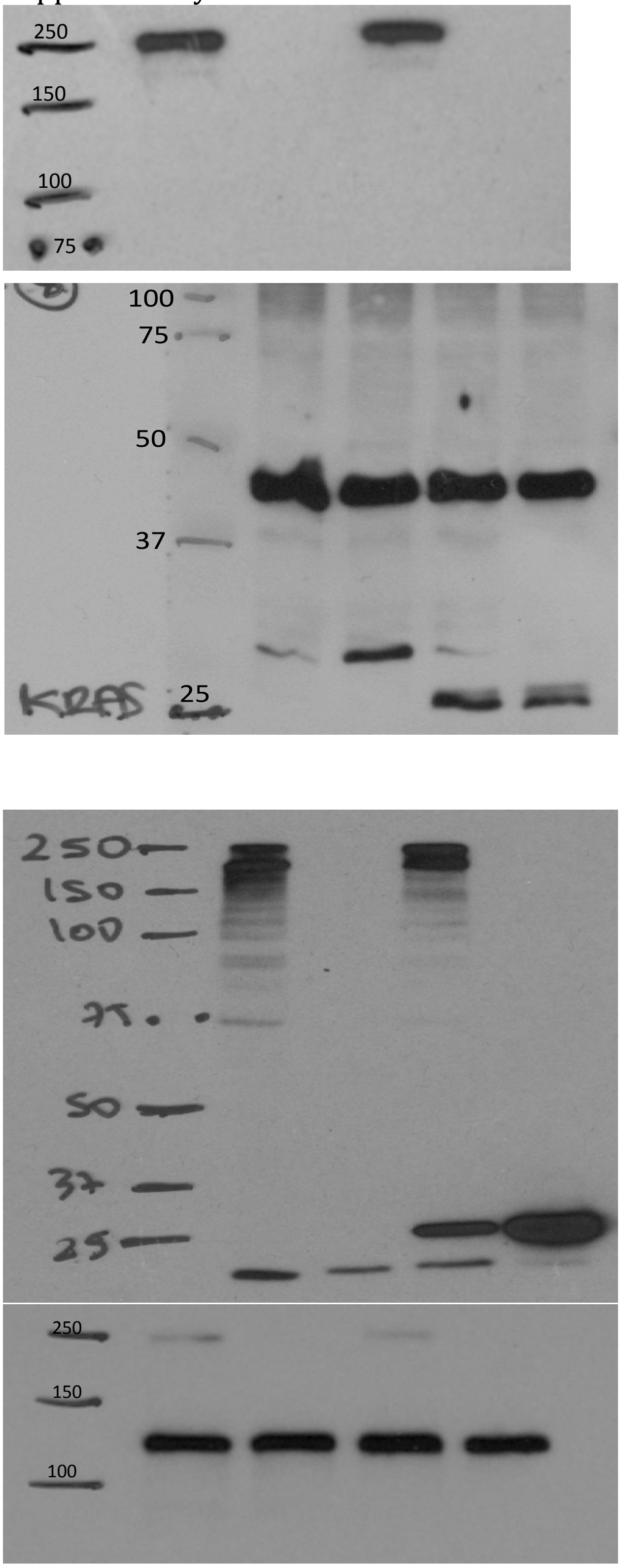

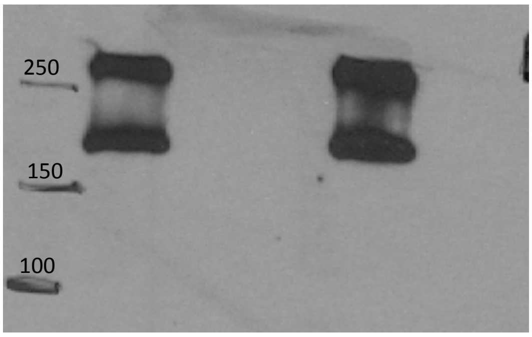
250
150
100

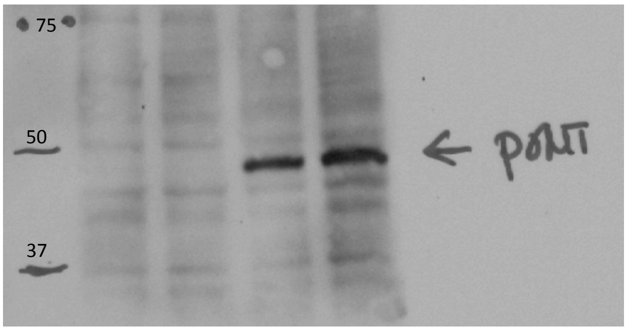
75
50
37
← pAKT

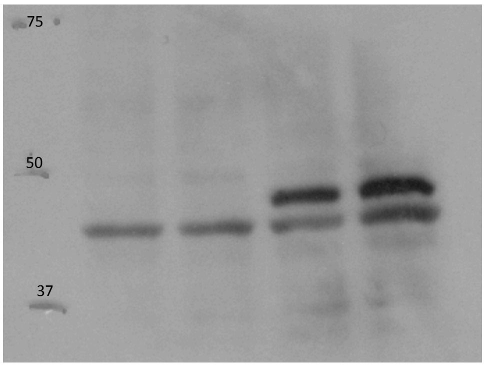
75
50
37

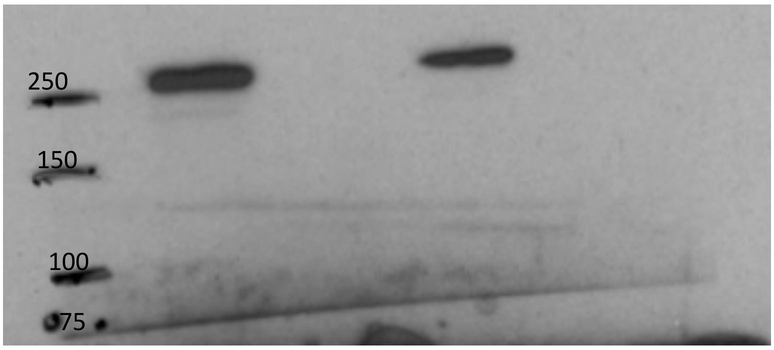
250
150
100
75

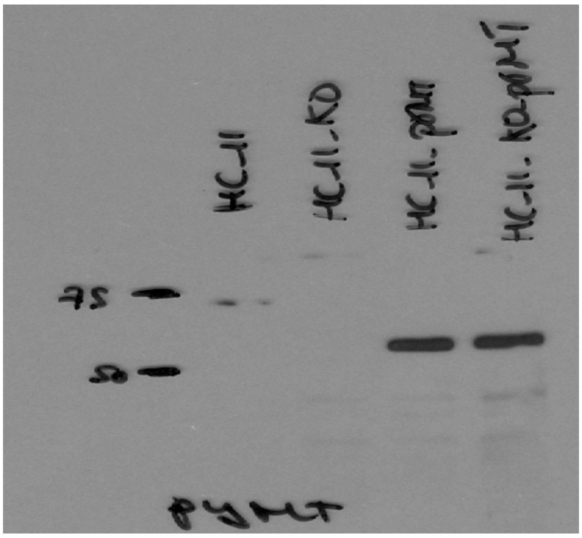
75
50
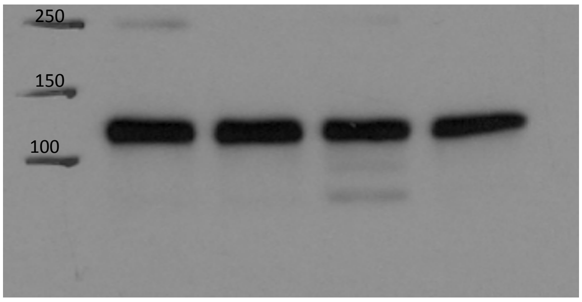
250
150
100
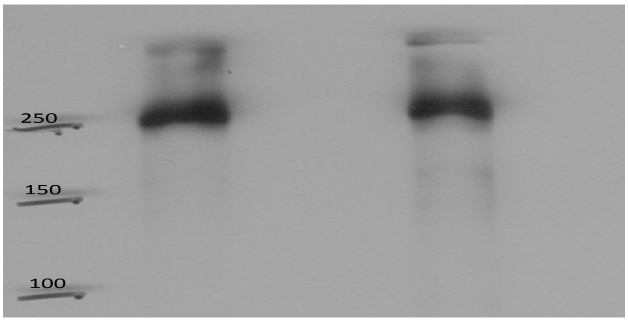
250
150
100
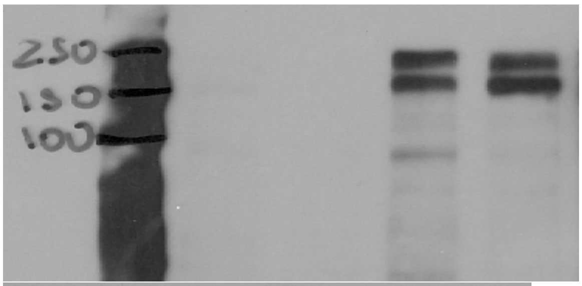
250
150
100
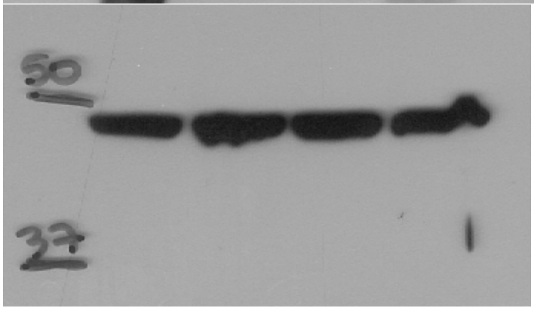
50
37

Supplementary Figure 2a

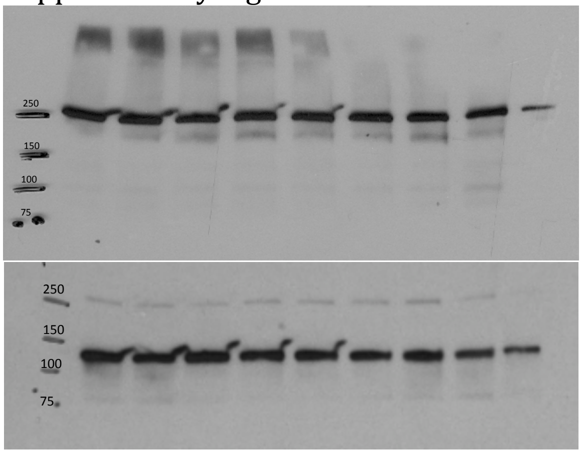

Supplementary Figure 2b

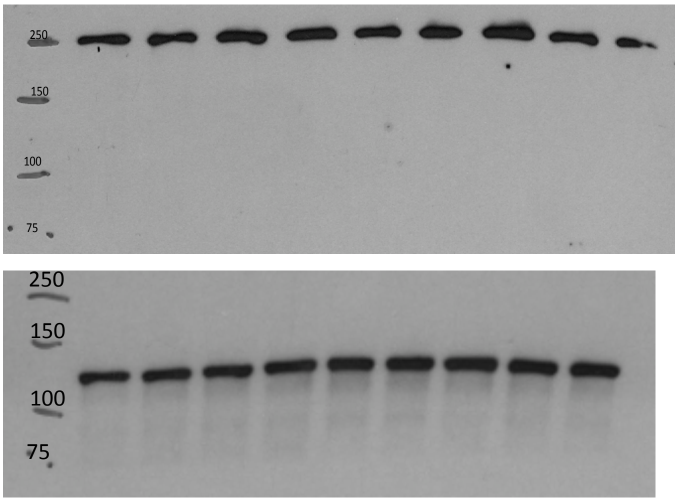

Supplementary Figure 2c

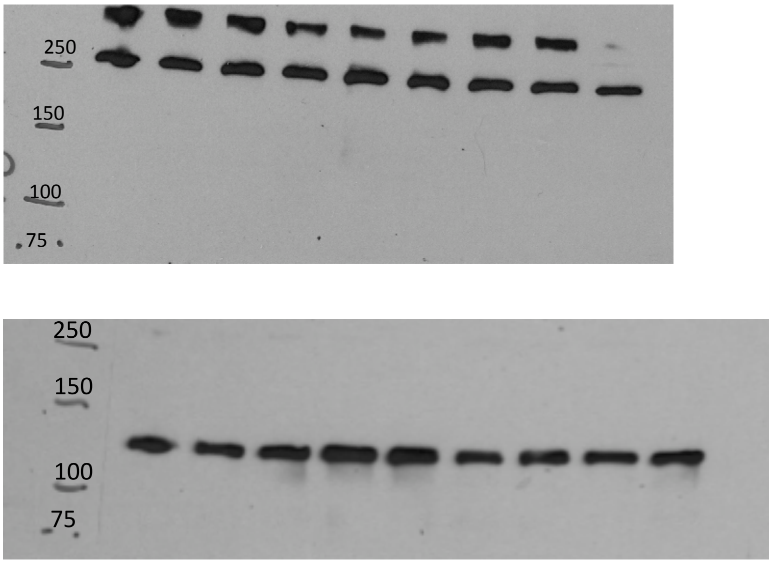

Supplementary Figure 2g

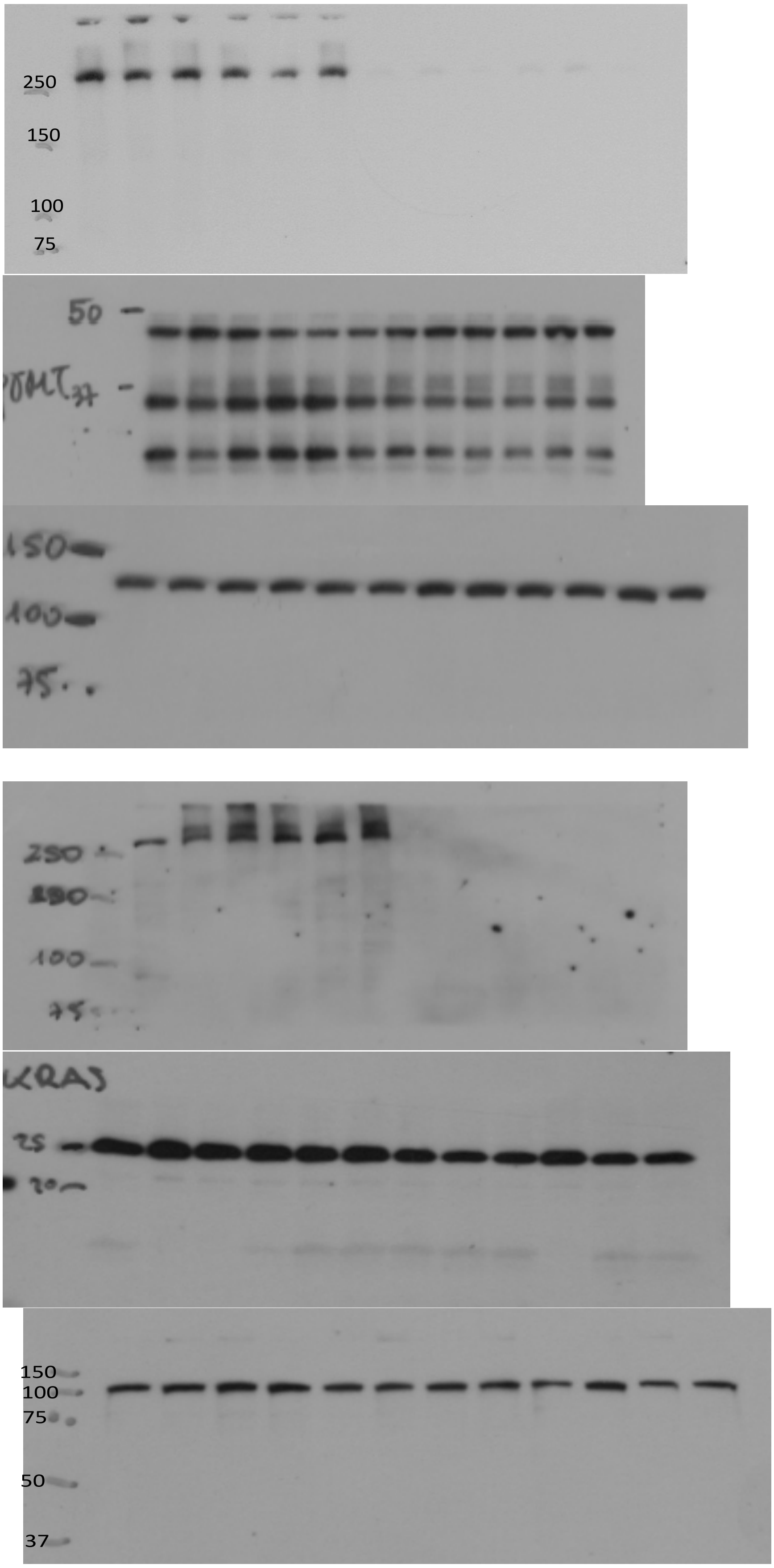

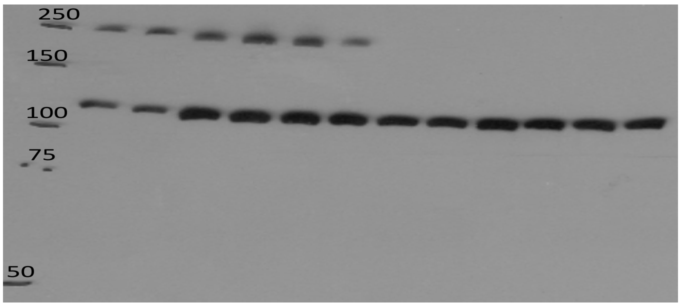

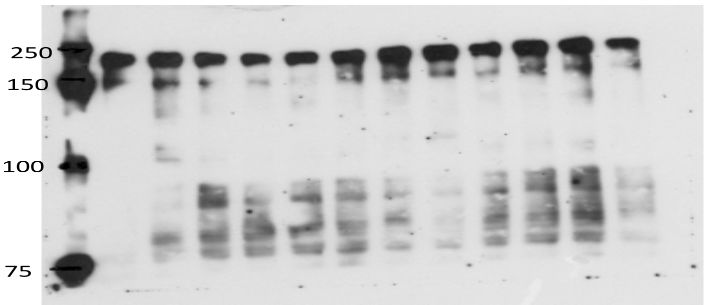

Supplementary Figure 7g

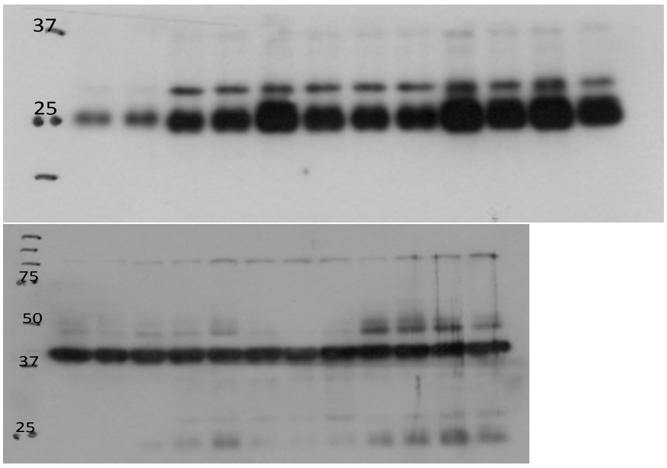

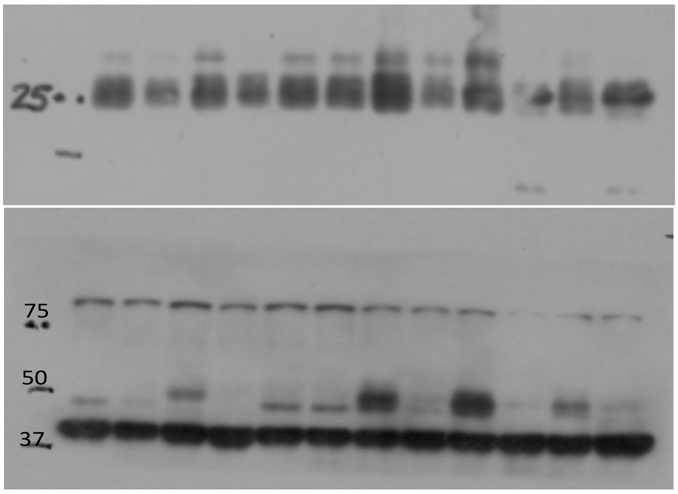

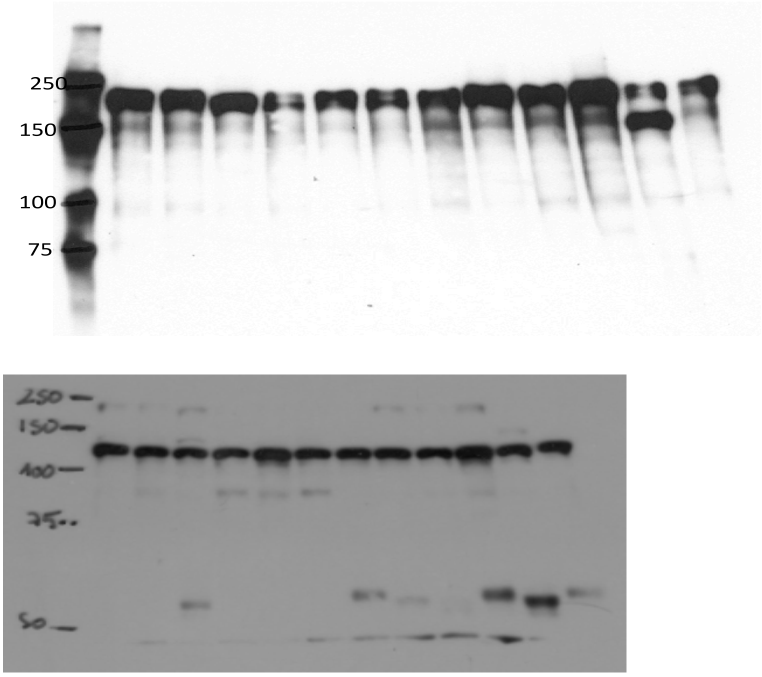

Supplementary Figure 10a

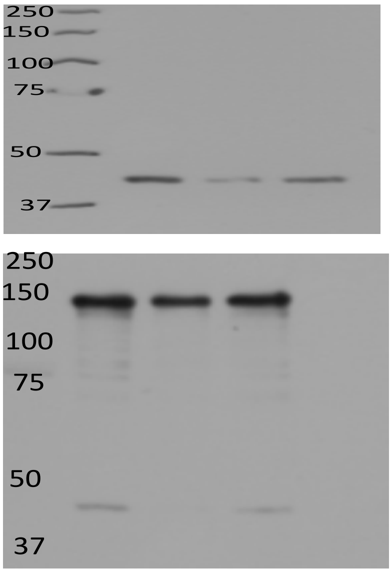

Supplement: Supplementary file 1 — Supplementary Information [file 41467_2019_13028_MOESM1_ESM.pdf]
