# Supplementary material for: Essentiality of fatty acid synthase in the 2D to anchorage-independent growth transition in transforming cells
Source: Nat Commun. 2019 Nov 1;10:5011. doi: 10.1038/s41467-019-13028-1 (PMC6825217; doi:10.1038/s41467-019-13028-1)
Supplement: Supplementary file 3 — Reporting Summary [file 41467_2019_13028_MOESM3_ESM.pdf]

nature research

Corresponding author(s): mquintela@cnio.es

Reporting Summary

Nature Research wishes to improve the reproducibility of the work that we publish. This form provides structure for consistency and transparency in reporting. For further information on Nature Research policies, see Authors & Referees and the Editorial Policy Checklist.

Statistical parameters

When statistical analyses are reported, confirm that the following items are present in the relevant location (e.g. figure legend, table legend, main text, or Methods section).

n/a	Confirmed	
☐	☒	The exact sample size (*n*) for each experimental group/condition, given as a discrete number and unit of measurement
☐	☒	An indication of whether measurements were taken from distinct samples or whether the same sample was measured repeatedly
☐	☒	The statistical test(s) used AND whether they are one- or two-sided<br>*Only common tests should be described solely by name; describe more complex techniques in the Methods section.*
☒	☐	A description of all covariates tested
☒	☐	A description of any assumptions or corrections, such as tests of normality and adjustment for multiple comparisons
☐	☒	A full description of the statistics including central tendency (e.g. means) or other basic estimates (e.g. regression coefficient) AND variation (e.g. standard deviation) or associated estimates of uncertainty (e.g. confidence intervals)
☒	☐	For null hypothesis testing, the test statistic (e.g. *F*, *t*, *r*) with confidence intervals, effect sizes, degrees of freedom and *P* value noted<br>*Give P values as exact values whenever suitable.*
☒	☐	For Bayesian analysis, information on the choice of priors and Markov chain Monte Carlo settings
☒	☐	For hierarchical and complex designs, identification of the appropriate level for tests and full reporting of outcomes
☒	☐	Estimates of effect sizes (e.g. Cohen's *d*, Pearson's *r*), indicating how they were calculated
☐	☒	Clearly defined error bars<br>*State explicitly what error bars represent (e.g. SD, SE, CI)*

Our web collection on statistics for biologists may be useful.

Software and code

Policy information about availability of computer code

Data collection	N/A
Data analysis	GraphPad Prism® Software<br>Kaplan-Meier survival curve: SPSS software (version 19)

For manuscripts utilizing custom algorithms or software that are central to the research but not yet described in published literature, software must be made available to editors/reviewers upon request. We strongly encourage code deposition in a community repository (e.g. GitHub). See the Nature Research guidelines for submitting code & software for further information.

Data

Policy information about availability of data

All manuscripts must include a data availability statement. This statement should provide the following information, where applicable:

- Accession codes, unique identifiers, or web links for publicly available datasets
- A list of figures that have associated raw data
- A description of any restrictions on data availability

Accesion codes: N/A

No restrictions on data availability. Data will be available upon manuscript acceptance.

Field-specific reporting

Please select the best fit for your research. If you are not sure, read the appropriate sections before making your selection.

- [x] Life sciences
- [ ] Behavioural & social sciences
- [ ] Ecological, evolutionary & environmental sciences

For a reference copy of the document with all sections, see nature.com/authors/policies/ReportingSummary-flat.pdf

Life sciences study design

All studies must disclose on these points even when the disclosure is negative.

Sample size	Sample size was chosen based on previous data.
Data exclusions	N/A
Replication	Replicates were done in all the experiments to verify the reproducibility of the experimental findings.
Randomization	N/A
Blinding	investigators were blinded to group assignment during the experiments.

Reporting for specific materials, systems and methods

Materials & experimental systems

n/a	Involved in the study	
☒	☐	Unique biological materials
☐	☒	Antibodies
☐	☒	Eukaryotic cell lines
☒	☐	Palaeontology
☐	☒	Animals and other organisms
☒	☐	Human research participants

Methods

n/a	Involved in the study	
☒	☐	ChIP-seq
☐	☒	Flow cytometry
☒	☐	MRI-based neuroimaging

Antibodies

Antibodies used	FASN(clone: C20G5; 3180, Cell Signaling), PyMT (NB100-2749, Novus Biological), K-Ras (clone: F234; sc-30; Santa Cruz), HER2 (2242, Cell Signaling), β-Actin (clone AC-15; A5441, Sigma), γ-tubulin (clone GTU-88; T6557, Sigma) and Vinculin (clone: hVIN-1;V9131, Sigma). anti-8-oxodG antibody (Clone: 2E2; 4354-MC-050, Trevigen). Alexa Fluor 594 donkey anti-mouse IgG (1:200; Thermo Fisher Scientific). Alexa Fluor 488 goat anti-mouse IgG (1:200; Thermo Fisher Scientific) and Alexa Fluor 555 goat antirabbit IgG (1:200; Thermo Fisher Scientific)
Validation	All the primary antibodies were validated. Validations are described on the manufacturer's websites.

Eukaryotic cell lines

Policy information about cell lines

Cell line source(s)	HC11 mammary epithelium: ATCC
Authentication	Cell lines from ATCC have been thoroughly tested and authenticated
Mycoplasma contamination	All cell lines were confirmed to be mycoplasma<br>479 negative by MycoalertTM Mycoplasma Detection Kit (Lonza).

Commonly misidentified lines (See ICLAC register) | N/A

Animals and other organisms

Policy information about studies involving animals; ARRIVE guidelines recommended for reporting animal research

Laboratory animals | athymic nude mice (Hsd: Athymic Nude-Foxn1nu), FASN lox/lox mice were generated from knockout embryonic stem cells (ES) for FASN (C57BL/6N-FASN tm1(KOMP)wtsi). Then mice were crossed to generate FVB pure background.

Wild animals | All animal experiments were approved by the Institutio de Salud Carlos Tercero Ethics Committee (PROEX/387/15) and performed in accordance with the guidelines stated in 21 the International Guiding Principles for Biomedical Research Involving Animals 559 developed by the Council for International Organizations of Medical Sciences.

Field-collected samples | N/A

Flow Cytometry

Plots

Confirm that:

☒ The axis labels state the marker and fluorochrome used (e.g. CD4-FITC).

☒ The axis scales are clearly visible. Include numbers along axes only for bottom left plot of group (a 'group' is an analysis of identical markers).

☒ All plots are contour plots with outliers or pseudocolor plots.

☒ A numerical value for number of cells or percentage (with statistics) is provided.

Methodology

Sample preparation | For apoptosis assay, MEFs were stained with DAPI and Annexin V (Cat no. 556547, FITC Annexin V Apoptosis Detection Kit I, BD Pharmingen™) at room temperature for 5 min and analyzed by flow cytometry using a FACSCalibur flow cytometer (FACScalibur, Becton-Dickinson). Mitochondria-mediated ROS generation was detected with the mitochondrial superoxide indicator MitoSOX-Red (Life technologies). For 2D measurement of mitochondrial ROS, MEFs and HC-11 cells were trypsinized and resuspended in DMEM and RPMI medium, 10% FBS. After centrifugation, cells were washed twice in PBS and stained with 3uM of MitoSOX-Red (Life technologies) for 20 min in PBS for 30 min at 37°C. Subsequently, the cells were washed twice in PBS followed by analysis on a FACS Calibur flow cytometer (Becton-Dickinson). For 3D measurement, MEFs and HC-11 cells were cultured in ultra-low attachment surface plates for at least 72h before ROS quantification. After incubation with MitoSOX-Red cells were then washed in PBS with 5mM EDTA to prevent aggregation of cells for FACS analysis.

Instrument | FACScalibur, Becton-Dickinson)

Software | Flowjo software (Tree Star, Ashland, OR).

Cell population abundance | N/A

Gating strategy | Gating strategy is provided in the Supplementary information

☒ Tick this box to confirm that a figure exemplifying the gating strategy is provided in the Supplementary Information.
